# Long-term dissemination of NDM-producing Enterobacterales in two French hospitals (2020–2024): outbreak complexity and the role of drains as environmental reservoirs

**DOI:** 10.1186/s13756-026-01766-z

**Published:** 2026-06-04

**Authors:** G. Ménard, D. Huart, K. Le Carrour-Quéré, F. Gourmelen, A. Morin Le Bihan, F. Guérin, L. Dortet, R. A. Bonnin, V. Cattoir, Cécile Emeraud

**Affiliations:** 1 Service of Bacteriology and Hospital Hygiene, CHU Rennes, Rennes, France; 2 UMR_S 1230 Inserm BRM, University of Rennes, Rennes, France; 3 Infection Prevention and Control Team, CH Saint-Malo, Saint Malo, France; 4 Associated French National Reference Centre for Antibiotic Resistance: Carbapenemase-Producing Enterobacterales, CHU Bicêtre, Le Kremlin-Bicêtre, France

**Keywords:** CPE, NDM, Whole-genome sequencing, Clonal transmission, Environmental reservoirs, Infection control

## Abstract

**Background:**

Carbapenemase-producing Enterobacterales (CPE) represent a major public health threat, particularly in healthcare settings where long-term outbreaks may be sustained by environmental reservoirs. In France, although OXA-48-like enzymes remain predominant, NDM-producing Enterobacterales are increasingly reported, notably among *Enterobacter cloacae* complex (ECC) and *Citrobacter freundii* complex (CFC). The contribution of hospital drainage systems to the persistence of such outbreaks remains insufficiently characterised.

**Methods:**

We conducted a five-year genomic and epidemiological investigation (2020–2024) of NDM-producing ECC and CFC in two hospitals in western France. Clinical and environmental isolates were analysed using whole-genome sequencing to assess clonality, resistance profiles and plasmid structures. Fourier-transform infrared spectroscopy was used to compare additional environmental and clinical isolates. Targeted environmental control bundles, including replacement with valve drains, daily bleaching and steam-based sanitization, were implemented and evaluated.

**Results:**

Fifty-one patients were identified as carriers or infected with NDM-producing ECC or CFC. Two clonal outbreaks were documented: *Enterobacter xiangfangensis* ST111, persisting in both hospitals over four years (*n* = 34), and *Citrobacter portucalensis* ST408, responsible for a shorter outbreak in one hospital (*n* = 7). Whole-genome sequencing confirmed clonal transmission without evidence of plasmid-mediated spread between species. Environmental sampling repeatedly identified *E. xiangfangensis* ST111 in drains, including sink and shared shower drains, over time, demonstrating sustained environmental persistence despite reinforced infection prevention and control measures.

**Conclusions:**

Our findings demonstrate that hospital drainage systems can act as long-term reservoirs sustaining the transmission of NDM-producing Enterobacterales. Standard infection control measures alone may be insufficient to control such outbreaks. Targeted environmental interventions should be systematically integrated into infection prevention strategies to limit the spread of highly resistant organisms.

**Supplementary Information:**

The online version contains supplementary material available at 10.1186/s13756-026-01766-z.

## Background

Carbapenems remain one of the last therapeutic options for treating infections caused by multidrug-resistant Enterobacterales [1]. However, resistance to these antibiotics is increasingly driven by the production of carbapenemase enzymes, which represent the main mechanisms of carbapenem resistance in these bacteria. Consequently, the global spread of carbapenemase-producing Enterobacterales (CPE) has become a major public health concern. Carbapenemases belong to three of the four Ambler classes: class A carbapenemases (mainly KPC types), class B carbapenemases or metallo-β-lactamases (MBLs), primarily NDM, VIM, and IMP types, and class D carbapenemases, predominantly OXA-48 type [2–4].

In France, OXA-48-like enzymes remain the most prevalent carbapenemases, despite an increase in NDM producers has been observed worldwide in recent years [5, 6]. Moreover, while carbapenemases in Enterobacterales have historically been found primarily in *Escherichia coli* and *Klebsiella pneumoniae*, *Enterobacter cloacae* complex (ECC) species and *Citrobacter freundii* complex (CFC) are now becoming increasingly prevalent, with many of these isolates producing MBLs. MBL-producing bacteria are difficult to treat since most new β-lactam/β-lactamase inhibitor combinations are ineffective [7, 8]. Cefiderocol and aztreonam-avibactam remain the most active options, though resistance is also emerging [9].

ECC and CFC isolates are frequently implicated in prolonged hospital outbreaks that persist over time [10–13], making them particularly challenging to eradicate. In addition to classical infection and prevention control (IPC) measures such as contact precautions and screening tests, recent studies have highlighted that long-term outbreaks may be linked to environmental reservoirs, the most commonly described being sanitary installations [11, 13–15]. Therefore, it is crucial to identify and characterise the mechanisms driving the persistence of these highly resistant, outbreak-causing bacteria in order to develop strategies to limit their spread. Such investigations are essential to inform infection control and national surveillance strategies.

Given the long-term increase of NDM-producing CPE, predominantly ECC and CFC isolates, in two facilities linked by regular patient transfers, we conducted an in-depth investigation of NDM-producing ECC and/or CFC isolates from patients and drains collected between 2020 and 2024 using whole genome sequencing (WGS). The aim was to determine whether these isolates reflected independent introduction events or the persistence of specific clones circulating multiple years, potentially maintained by environmental reservoirs.

## Methods

### Context and study design

The study was conducted in two healthcare facilities in Western France: the 1900-bed University Hospital of Rennes (UHR) and the 700-bed regional hospital of Saint-Malo (RHSM). In July 2023, a follow-up was initiated due to an increase in NDM-CPE cases, outbreak episodes, and frequent patient transfers between the two healthcare facilities. Consequently, we investigated retrospectively then prospectively all NDM-CPE cases from January 2020 to December 2024, to characterise and track the dissemination of NDM-producing ECC and CFC.

### Case definitions

Cases were defined as patients colonised and/or infected by NDM-producing ECC and/or CFC. Colonisation was defined by a positive rectal screening whereas infection was defined with at least one positive clinical sample. CPE isolates were considered as (i) imported if recovered before admission or during the first 48 h of hospital stay, (ii) hospital-acquired if identified after 48 h with a previously negative sample; or (iii) undefined if detected after 48 h without prior sampling. Contact cases were identified according to French national guidelines [16], i.e. patients hospitalized in the same ward concurrently with a positive patient. A species-related outbreak was defined as the detection of at least two NDM-producing ECC or CFC isolates in two epidemiologically-linked patients, whereas a plasmid-mediated outbreak was defined as the detection of NDM-producing ECC and CFC isolates sharing the same plasmid.

### Infection prevention and control measures

In both facilities, French national infection control guidelines issued by the *Haut Conseil de la Santé Publique* were followed [16]. Briefly, patients with confirmed colonisation or infection were placed in single rooms, and contact precautions were systematically applied. Whenever feasible, the use of shared toilets and showers was restricted for positive patients. Rectal screening of contact cases was also systematically performed.

During outbreak situations, measures were reinforced through enhanced monitoring of contact cases and, when necessary and possible, the deployment of a dedicated nursing team.

Daily environmental cleaning was performed using detergent-disinfectants based on peracetic acid, also containing low concentrations of quaternary ammonium compounds (Anios Oxy’Floor^®^), or on quaternary ammonium compounds combined with amines (Surfanios^®^), according to local protocols (RHSM and UHR).

### Environmental sampling

During the five-year study period, environmental sampling of drains was performed on a targeted basis to identify potential reservoirs during uncontrolled outbreaks and to evaluate the effectiveness of environmental disinfection strategies, including bleach treatment, steam-based sanitisation, and installation of valve drains. The frequency of sampling varied according to the epidemiological situation and the stage of implementation and evaluation of corrective control measures. Drains selected for sampling included those located in rooms of colonised or infected patients, shared sanitary facilities (shower drains), as well as in some cases, rooms without known carriers. The bleach treatment consisted of applying 0.1% bleach daily for 15 min to drains equipped with valves.

We focused on three distinct bundles, depending on the facility and the type of clinical ward (Table 1). Implementation periods and target wards varied between facilities according to outbreak dynamics.

**Table 1:** Summary of environmental control bundles implemented in both facilities (2020–2024).

Bundle	Facility/Ward	Period	Main control measures
A	RHSM—Ward A (Intensive care unit)	2020–2024	Longitudinal follow-up of sink drains: replacement of conventional drains with valve drains; daily bleaching, installation of plexiglass barriers between the worktop and the sink, steam sanitisation of drains at patient exit.
B	RHSM—(multiple wards)	Feb–Mar 2024	Targeted drain sampling before and after steam sanitisation (sinks, showers) in rooms previously occupied by positive patients.
C	UHR—Ward F1 (Digestive surgery)	2024	Longitudinal follow-up of sink and shared shower drains: replacement of conventional sink drains with valve drains; daily bleaching; single-shot steam sanitisation; use of Anios OXY’FLOOR detergent-disinfectant for shared shower drains.

RHSM: Regional Hospital of Saint-Malo; UHR: University Hospital of Rennes

Samples were collected using cotton swabs, discharged in tryptone-soy liquid enrichment broth (Oxoid) and incubated for 24 h at 37 °C. Turbid broths were inoculated on ChromID carba Smart (bioMérieux, Marcy l’Etoile, France) and incubated for 48 h at 37 °C.

### Microbial investigations

Rectal screening swabs were plated on ChromID carba smart agar and incubated for 24 h at 37 °C. Bacterial identification was performed using matrix-assisted laser desorption/ionization-time-of flight mass spectrometry (MALDI-TOF, Biotyper V12; Bruker, Billerica, MA, USA). Noteworthy, this method lacks of discriminatory power to distinguish between different ECC and CFC species [17], and then we used the classification proposed by Wu et al. [18] based on WGS data. Carbapenemase production was confirmed either by real-time PCR (Xpert Carba-R assay, GenExpert Cepheid, USA) or by immunochromatographic assay (NG-CARBA 5, NG Biotech, Guipry, France), both detecting the five main carbapenemase families: OXA-48-like, NDM, VIM, IMP and KPC. All clinical NDM-producing isolates (ECC and CFC) and a representative subset of environmental CPE isolates (*n* = 5) were sent to the French National Reference Centre (F-NRC) for further characterisation. Other environmental CPE isolates were typed using Fourier transform infrared (FTIR) spectroscopy (IR Biotyper, Bruker Daltonics GmbH & Co. KG, Bremen, Germany) as previously described [19, 20]. To define groups, that is, related isolates, we used the cutoff value provided by the manufacturer (0.15–0.20) and the results obtained with internal controls to consider both intra- and interplate variations. The cutoff value referred to which distance the spectra were in the same group. To compare clinical and environmental strains, a subset of clinical isolates with known sequence types (STs) was included.

### Antimicrobial susceptibility testing

Minimal inhibitory concentrations (MICs) to critical and last-resort antibiotics were performed using EUMDROXF Sensititre™ broth microdilution plate manufactured by Thermo Scientific™ (ThermoFisher Scientific, Dardilly, France). Cefiderocol MICs were obtained using UMIC^®^ broth microdilution strips (Brucker Daltonics GmbH & Co. KG, Bremen, Germany). MICs for these last-resort antibiotics were performed on a panel of representative clinical isolates of *E. xiangfangensis* ST111 (*n* = 21) and *C. portucalensis* ST408 (*n* = 5). MICs were interpreted according to EUCAST clinical breakpoints for Enterobacterales.

### Whole-genome sequencing and phylogenetic analysis

A short-read next-generation sequencing was performed on all strains included in the study using the NovaSeq 6000 system (Illumina^®^, Genbank accession Number PRJNA1353879). Reads were assembled using Shovill v1.1.0 and SPAdes v3.14.0. MLST, resistome analyses and plasmid content were carried out using the pubMLST, ResFinder and PlasmidFinder databases, respectively. Furthermore, to reconstruct and characterise the plasmids carrying the *bla*_NDM−1_ gene, based on the initial analyses performed on the Illumina^®^ sequencing data, we selected one representative strains of *E. xiangfangensis* ST111 and *C. portucalensis* ST408 for long-read sequencing using the MinION technology (Oxford Nanopore). MinION long-reads were assembled using Trycycler v0.5.5. and corrected with Illumina^®^ reads using Polypolish v0.6.0.

For phylogenetic single-nucleotide polymorphism (SNP)-based analysis, NGS sequence reads were mapped to a reference genome included in the study (*E. xiangfangensis* 253D10 and *C. portucalensis* 406G7) using SNIppy v4.6.0. To further investigate the clonality of strains within *E. xiangfangensis* ST111 and *C. portucalensis* ST408, SNP matrices were constructed. Metadata and phylogenetic trees were visualised using iTOL v6.5.2. Using MinION sequencing, we reconstructed the various plasmids present in these strains. The plasmids, as well as the genetic environments surrounding the carbapenemase-encoding genes, were annotated using RAST, NCBI tools, and CLC workbench v21 software. ECC and CFC species were determined by phylogeny using WGS data compared to a large collection of reference genomes.

## Results

### Case characteristics

A total of 51 patients (13 in RHSM and 38 in UHR) positive for NDM-producing CPE colonisation or infection were identified during the five-year study period (Additional Figure S1). Among them, 38 patients were positive for NDM-producing ECC (13 in RHSM and 25 in UHR) while 13 patients were positive for NDM-producing CFC (only in UHR).

Between April 2020 and February 2023, nine cases were only described in RHSM, and mainly related to an outbreak in a polyvalent intensive care unit (ward A). Thereafter, a shift was observed, with most cases (82%) identified in UHR, including a large presumed outbreak in digestive surgery units (wards F1 and F2), involving both ECC and CFC isolates.

Demographic and clinical data are provided in Additional Table 1. Of the 51 patients, 29 were colonised during the study, including 16 ECC and all *C. freundii* cases. In contrast, 22 infections were observed, all of them caused by ECC isolates, including 12 bacteremia. Six cases were classified as imported, including two patients previously hospitalised abroad (< 1 year) and one hospitalized in the two facilities. Twenty-five cases were considered as hospital acquired, and 20 classified as undefined, despite documented contact with positive cases for 4 patients. Notably, six patients were hospitalised in both facilities during their care pathways, and 31 patients had used shared showers.

### Clonality and resistance gene profiles of ECC and CFC isolates

Among the 38 non-duplicate ECC isolates sent to the F-NRC, species identification by WGS and MLST analysis identified 34 *E. xiangfangensis* ST111, two additional *E. xiangfangensis* (ST1053 and ST728), one *E. hoffmannii* ST97, and one *E. hormaechei* ST269. All isolates were identified as NDM-1 producers.

**Fig. 1:**
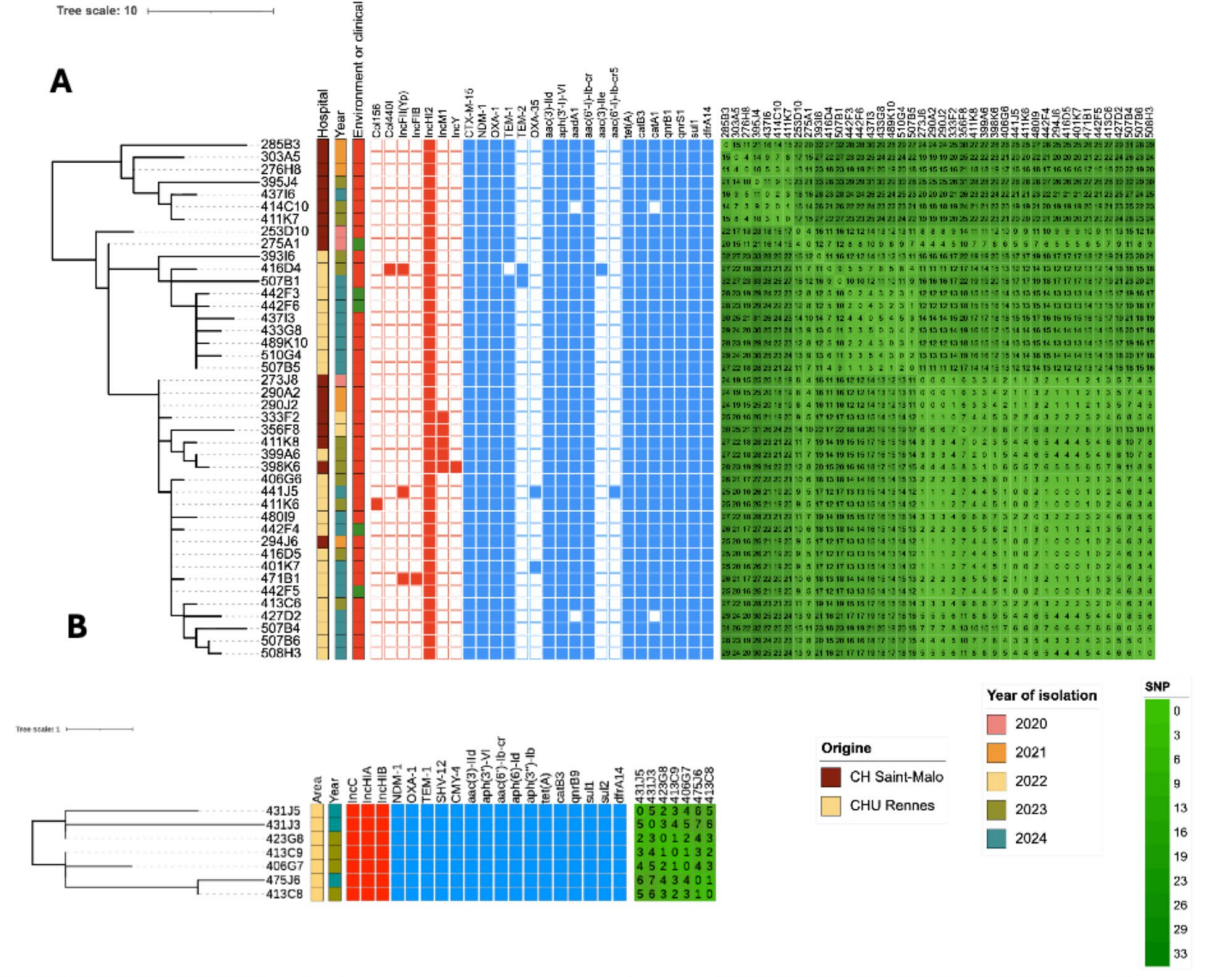
Phylogenetic analysis, resistome, plasmid content, and SNP distances among the outbreak isolates. **A** *E. xiangfangensis* ST111 and **B** *C. portucalensis* ST408 isolates. The phylogenetic trees were constructed based on core genome single-nucleotide polymorphism (SNP) alignments. The corresponding heatmaps display the pairwise SNP distances between isolates

To assess clonal dissemination within the ST111 group, a SNP matrix was constructed, revealing a maximum of 35 SNPs between two isolates (Fig. 1A). Plasmid analysis showed that all *E. xiangfangensis* ST111 strains harbored an IncHI2-type plasmid, with a subset also carrying additional plasmid types (Fig. 1A). Regarding acquired resistance genes, these strains exhibited a consistent profile, including β-lactam resistance genes other than *bla*_NDM−1_ (*bla*_CTX−M−15_, *bla*_OXA−1_, *bla*_TEM−1_) as well as genes conferring resistance to aminoglycosides, tetracyclines, quinolones, and trimethoprim-sulfamethoxazole (Fig. 1A).

Concerning the 13 CFC isolates, seven belonged to the ST408 (54%), three to the ST64 (23%) and three sporadic isolates not related as ST22, ST98 and ST62, respectively. The ST408 isolates initially identified as *C. freundii* using MALDI-TOF technology were reclassified as *C. portucalensis* after WGS analysis, and all strains produced NDM-1. A maximum of only 6 SNPs between two strains was observed (Fig. 1B). Plasmid analysis revealed that all ST408 strains harbored the same plasmid types (IncC, IncHI1, and IncHIB) and shared various resistance genes, including β-lactamase (*bla*_OXA−1_, *bla*_TEM−1_, *bla*_SHV−12_, *bla*_CMY−4_, and *bla*_CMY−2_) and other resistance genes (Fig. 1B).

### Epidemiological investigation and transmission dynamics of the epidemic clones

**Fig. 2:**
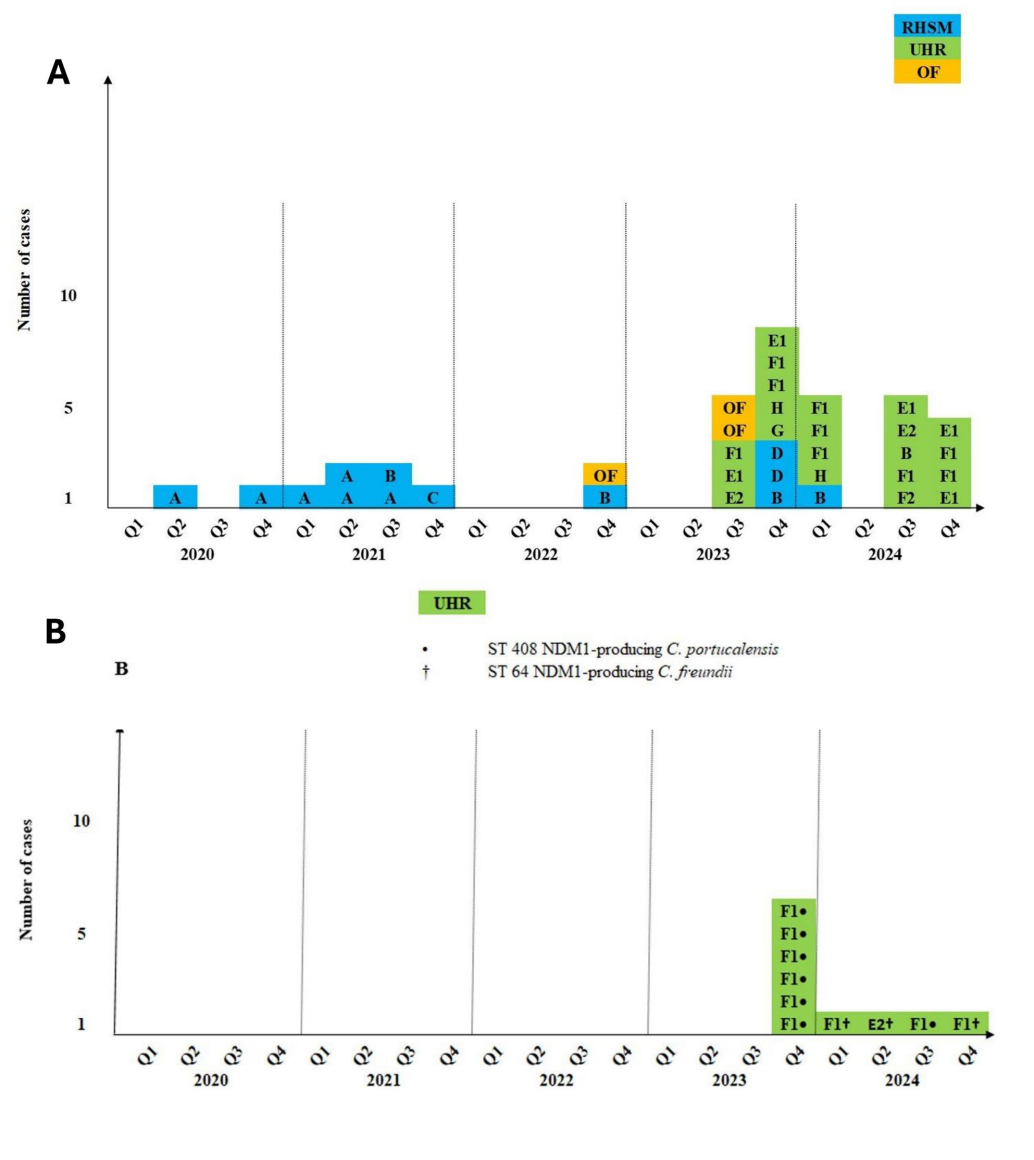
Temporal distribution of patients carrying or infected with *Enterobacter cloacae* complex (ECC) ST111 and *Citrobacter freundii* complex (CFC) isolates in the Regional Hospital of Saint-Malo (RHSM), the University Hospital of Rennes (UHR). **A** Bars represent the quarterly number of patients carrying or infected with *E. xiangfangensis* ST111. Colours indicate the different healthcare facilities involved: blue for RHSM, green for UHR, and orange for OF (outside facility). Each letter within a bar refers to a specific ward, as detailed below. The three OF cases were retrospectively identified by the French National Reference Centre (F-NRC) as clonally related to the RHSM outbreak, with no initial screening performed at the time of detection. **B** Bars represent the quarterly number of patients carrying or infected with *C. freundii* complex isolates. Data correspond to UHR only. Dots (•) and crosses (†) denote sequence types ST408 and ST64, respectively, both producing NDM-1 carbapenemase. Letters refer to wards, as described in panel (A). Ward codes: A = Polyvalent Intensive Care Unit; B = Infectious Diseases; C = Haematology; D = Geriatrics; E1 = Hepatology-A; E2 = Hepatology-B; F1 = Digestive Surgery-A; F2 = Digestive Surgery-B; G = Surgical Intensive Care Unit; H = Urology; I = Cardiac Intensive Care Unit

WGS analysis revealed that a clone of NDM-producing *E. xiangfangensis* ST111 persisted and disseminated across both facilities without clear epidemiological connection (Fig. 2A). The first case was described in April 2020 at the RHSM and classified as hospital acquired. No secondary cases were detected following rectal screening until December 2020, when five sporadic cases emerged up to July 2021 in the same ward (ward A). Subsequently, four additional cases were identified between 2021 and 2024 in ward B, followed by two closely related cases in ward D in 2023. At UHR, the first case appeared in July 2023, with no epidemiological links to RHSM and without evidence of cross transmission (ward E2). The second case in August 2023 was connected to the RHSM since the patient was recently transferred from the RHSM (ward E1). Investigations have not revealed any other cases. In September 2023, a patient was admitted in a digestive surgical ward (ward F1) for an infection caused by the ST111 *E. xiangfangensis* clone, the case was considered imported in accordance with our criteria. After that, 12 cases (out of 18) were reported in four connected wards (ward F1, F2, G and H) spanning from October 2023 to December 2024. Six other cases were detected in hepatology wards (wards E1 and E2) between November 2023 and December 2024 and one in infectious disease. Interestingly, according to the national surveillance data from the F-NRC, this *E. xiangfangensis* ST111 clone was exclusively identified in our two facilities. No other cases were reported elsewhere in France, except for three retrospective cases linked to prior hospitalisations at RHSM, where no microbial screening had been performed.

Regarding *C. portucalensis*, WGS analysis confirmed a true outbreak involving the ST408 clone (Fig. 2B). This outbreak occurred over a short period between October and December 2023 in the digestive surgical ward (ward F1), with one additional case identified later in July 2024 in the same ward. Finally, three *C. freundii* ST64 isolates were confirmed as hospital-acquired and clonally related, although the corresponding patients were hospitalised at different times and did not overlap.

To note, high levels of antimicrobial resistance of NDM-1 producing ST111 *E. xiangfangensis* and ST408 *C. portucalensis* isolates were demonstrated. The only antibiotics that retained activity against these strains were colistin and the aztreonam-avibactam combination (Additional Table 2).

### Characterisation of bla_NDM−1_-carrying plasmids in E. xiangfangensis ST111 and C. portucalensisST408 isolates

To reconstruct the plasmid carrying the *bla*_NDM−1_ gene, long-read sequencing was performed using MinION technology on a representative strain from each group (strain 433G8 for *E. xiangfangensis* ST111 and strain 406G7 for *C. portucalensis* ST408).

**Fig. 3:**
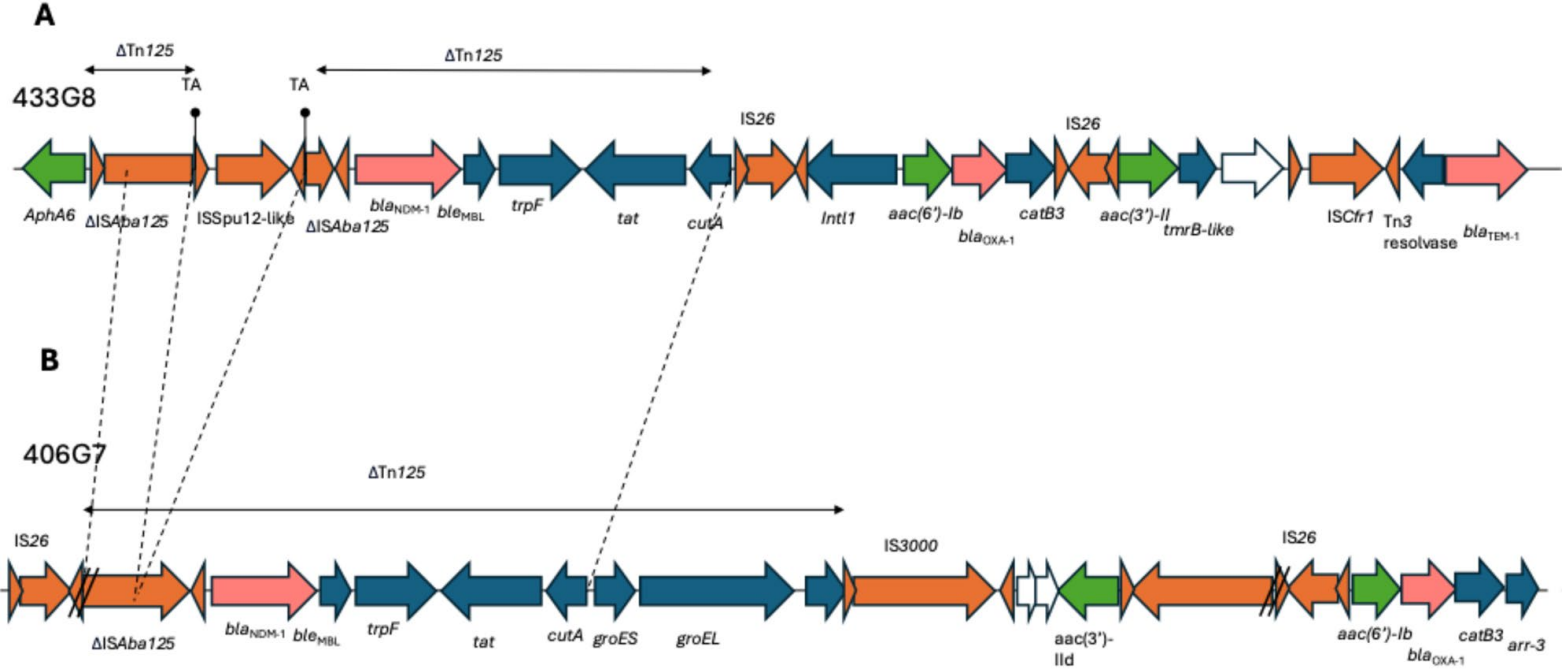
Genetic environments of the bla_NDM−1_ gene in **A** *E. xiangfangensis* ST111 (strain 433G8) and **B** *C. portucalensis* ST408 (strain 406G7), reconstructed using Nanopore long-read sequencing. Genes encoding β-lactamases are shown in pink, aminoglycoside resistance genes in green, insertion sequences in orange, and other genes in blue

In the 433G8 *E. xiangfangensis* ST111 strain, a single IncHI2-type plasmid of 245,680 bp was detected, harboring other β-lactam resistance genes (*bla*_CTX-M-15_, *bla*_OXA-1_, and *bla*_TEM-1_). This plasmid carries all the common resistance genes found across the ST111 strains, as shown in Fig. 3A, except in strain 416D4, which harbored *bla*_TEM-2_ instead of *bla*_TEM-1_. Illumina short reads from this strain mapped perfectly to the reconstructed plasmid obtained from strain 433G8, confirming that this was a single point mutation within an otherwise identical plasmid backbone. The close genetic environments of the *bla*_NDM-1_ gene were analysed for both clones. In isolate 433G8, upstream of the *bla*_NDM-1_ gene, a copy of IS*Aba125* was identified, which provides promoter sequences facilitating *bla*_NDM-1_ expression [21]. This IS*Aba125* sequence was truncated by an insertion of an IS*Spu12*-like element, with a 2-bp target site duplication (TA), suggesting a transposition process. Downstream of *bla*_NDM-1_, a bleomycin resistance gene (*ble*_MBL_) followed by *trpF*, tat, and *cutA* genes was present. These genes form part of the transposon Tn*125*, originally responsible for the dissemination of *bla*_NDM-1_ [22]. However, the 3’ end of Tn*125* was truncated by a copy of IS*26*, downstream of which a class 1 integron containing three resistance genes was identified, followed by another IS*26* copy (Fig. 3A).

For *C. portucalensis* strain 406G7, two plasmids were identified: one IncC-type plasmid of 206,629 base pairs, carrying *bla*_NDM−1_ and all other resistance genes associated with this strain group, and a second plasmid of 300,911 base pairs, carrying both IncHIA and IncHIB plasmid types but without any additional resistance genes. To confirm plasmid identity across all strains, reads were mapped against the reconstructed plasmids, verifying the plasmid structures within each respective group. In isolate 406G7, the *bla*_NDM−1_ gene was also associated with a remnant of Tn*125*. Upstream, a truncated copy of IS*Aba125* was found, interrupted by IS*26*. Downstream of the *bla*_NDM−1_ gene, Tn*125* was truncated at its 3’ end by IS*3000*, followed by *aac*(3’)-IId and an additional copy of IS*26*, which was adjacent to a class 1 integron. This integron carried the same gene array as that found in 433G8, including *aac*(6’)-Ib, *bla*_OXA−1_, and *catB3* (Fig. 3B).

### Environmental control measures for the eviction of these strains: outcomes and effectiveness

A total of 291 environmental samples were collected across both sites, of which 26 (9%) tested positive. These included seven NDM-producing ECC isolates from RHSM, as well as eight NDM-producing ECC, six VIM-producing ECC, and five NDM-producing CFC isolates from UHR. Notably, approximately 50% of the positive samples were obtained from shower drains, mostly shared ones (Additional Table 1**)**.

Two typing methods were used to assess relatedness between clinical and environmental isolates. WGS confirmed the clonality of a subset of environmental NDM-producing ECC isolates—one from RHSM (from 2020) and four from UHR—all belonging to the ST111. Additional environmental strains were analysed using FTIR spectroscopy, providing further insights. Among environmental ECC isolates (Additional Table 1, Additional Fig. 2**)**, 13 of 16 NDM-producing strains clustered with patient-derived ST111 isolates. Although no clear spatial or temporal link could be established between positive sink drains and specific patient rooms, most affected patients had documented exposure to shared showers during their hospital stay. However, the temporal relationship between exposure and acquisition could not be systematically established, and this observation should therefore be interpreted as a potential, rather than confirmed, source of transmission.

For CFC isolates (Additional Table 1, Additional Fig. 3**)**: only one sink-derived strain clustered with *C. portucalensis* ST408 patient isolates. No epidemiological data supported a link between this environmental isolate and a patient who tested positive in the corresponding room. The four remaining environmental CFC isolates could not be epidemiologically linked to specific patients, despite persistent contamination in shared showers in the affected ward.

**Fig. 4:**
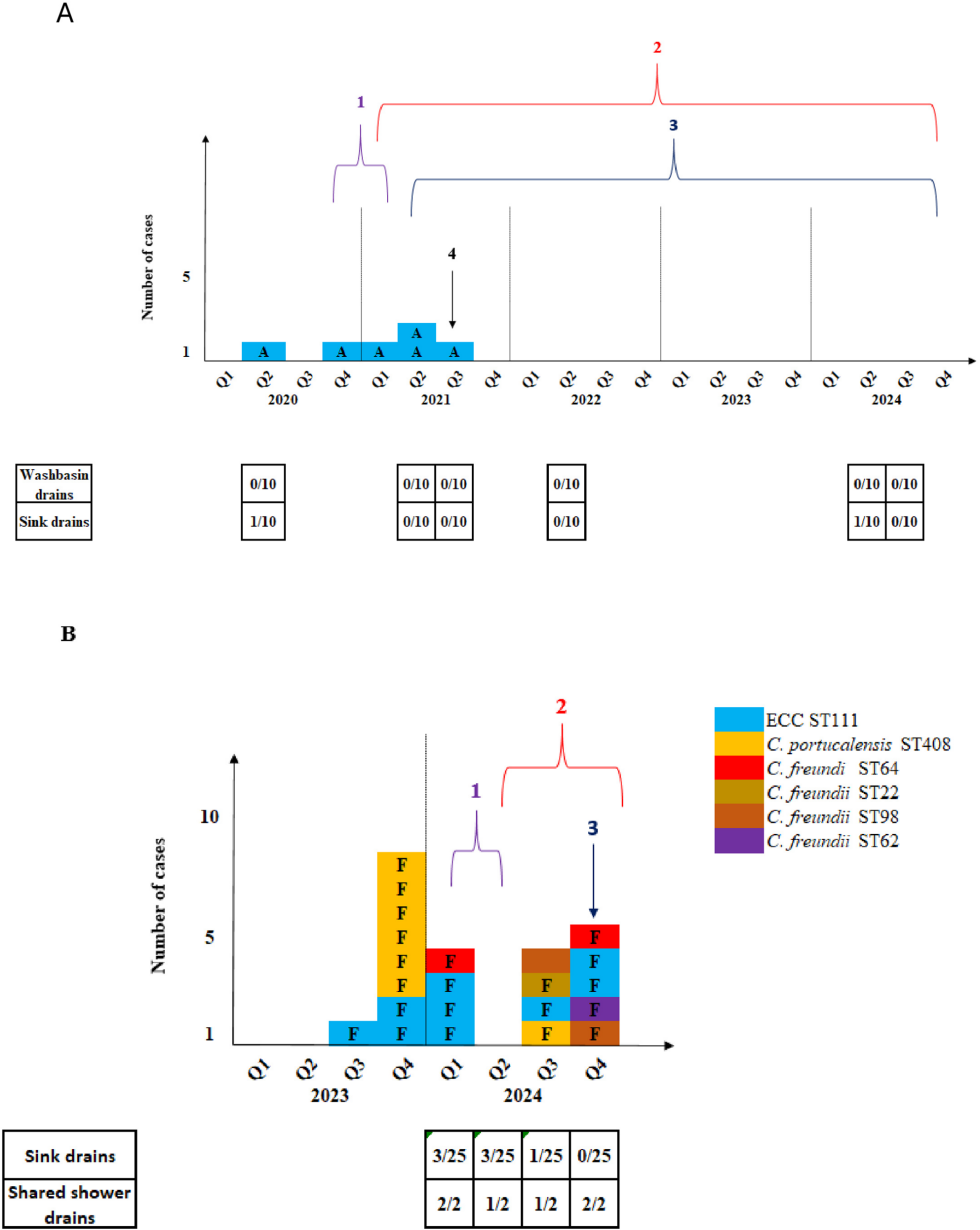
Temporal distribution of CPE cases and corresponding environmental sampling before and after implementation of environmental control bundles in RHSM and UHR. **A** Bars represent the quarterly number of patients carrying or infected with *CPE* at the Regional Hospital of Saint-Malo (RHSM). The letter “A” refers to ward A, where the *E. xiangfangensis* ST111 outbreak occurred. Brackets (1–4) correspond to successive environmental interventions: (1) replacement of conventional drains with valve drains, (2) daily bleaching, (3) steam sanitisation of drains at patient discharge, and (4) installation of plexiglass barriers between clean and wastewater points. **B** Bars represent the quarterly number of patients carrying or infected with ECC and CFC isolates at the University Hospital of Rennes (UHR). Colours indicate the different sequence types (STs) identified among ECC and CFC isolates, including *E. xiangfangensis* ST111 and *C. portucalensis* ST408. The letter “F” refers to ward F (digestive surgery). Brackets (1–3) correspond to the successive implementation of Bundle C measures: (1) replacement of conventional sink drains with valve drains, (2) daily bleaching, and (3) single-shot steam sanitization. Results of environmental sampling (sink drains) are shown below the graph as number of positive samples / total tested, including all CPE

Given the persistence of NDM-producing ECC and CFC despite standard IPC measures, the intervention focused on environmental sources suspected to contribute to transmission. In wards with ongoing transmission, targeted actions were progressively implemented: daily disinfection of drains using 0.1% bleach, steam sanitisation of sinks and showers, replacement of conventional drains with valve drains, and installation of splash barriers around sinks The effectiveness of the various control measures differed between the two facilities. At RHSM, both the bundles A and B (Fig. 4A; Table 2 were successful since only one sample was positive (likely due to suboptimal daily bleaching practices). In contrast, results at UHR with bundle C were less satisfactory, highlighting the failure to eliminate certain hotspots, particularly shared shower drains (Fig. 4B).

**Table 2:** *Drain sampling results by ward before and after steam sanitisation (Bundle B*,* RHSM*,* 2024)*

	February 2024 (before steam sanitisation)		March 2024 (after steam sanitisation)	
	Sink drains	Shower drains	Sink drains	shower drains
Infectious Diseases (ward B)	0/11	1/3*	–	0/1
Haematology (ward C)	0/6	1/4*	–	0/1
Geriatrics (ward D)	1/4	2/5*	0/1	0/2

*including share-shower drains. Only positive results were re-tested after steam sanitisation

## Discussion

This study reports a marked increase in NDM-1-producing Enterobacterales in two hospitals with previously low endemic levels. Using WGS, we identified the emergence and persistence of two epidemic clones: *E. xiangfangensis* ST111, circulating across both facilities from 2020 to 2024, and *C. portucalensis* ST408, responsible for a shorter outbreak in UHR in 2023. These clones, which have not been reported elsewhere in France, appear to be locally established within our facilities. In response, inter-facility communication and alert systems were strengthened to improve IPC.

Two mechanisms were investigated to explain the epidemiology: (i) plasmid-mediated transmission, and (ii) the contribution of a persistent environmental reservoir. WGS revealed that both outbreaks were caused by a single clone in each species. For *C. portucalensis*, this conclusion was strongly supported by the low number of SNPs observed (fewer than 6 SNPs between isolates). For *E. xiangfangensis* ST111, the maximum of 35 SNPs observed between isolates suggests clonal spread, even though a formal SNP threshold for defining clonal relationships is lacking. Moreover, the reconstruction of the *bla*_NDM−1_-carrying plasmids using long-read sequencing demonstrated that these two outbreaks were independent, as the plasmids identified in *E. xiangfangensis* ST111 and *C. portucalensis* ST408 were completely different between the two species.

Although IPC measures -such as hand hygiene compliance and appropriate use of personal protective equipment (PPE) -remain critical, the long-lasting persistence of the *E. xiangfangensis* ST111 clone suggests an environmental reservoir. Hospital sanitary installations, notably drains, have previously been identified as potential reservoirs [11–13, 23–25].

The *E. xiangfangensis* ST111 clone was frequently recovered from drain samples collected between 2020 and 2024 at both facilities, in contrast to the more transient nature of the *C. portucalensis* ST408 outbreak. Cross-contamination between patients and hospital sanitary installations is bidirectional, with transmissions more frequently occurring from patients to the environment [24]. In most cases, no direct evidence supported transmission from drains to patients was found, particularly for washbasin and sink drains, where contamination more frequently reflected prior or concurrent occupancy by known carriers. Interestingly, *E. xiangfangensis* ST111 clone was detected in some drain samples from rooms without known carriers, but not in plumbing samples (data not shown), possibly due to methodological differences in processing, with enrichment performed only for environmental samples.

Shared shower drains, used by a large proportion of patients, were frequently contaminated with *E. xiangfangensis* ST111 from 2020 to 2024, some of them sustainably, corroborating previous reports of high levels of CPE contamination in shared showers [12]. These findings suggest their role as persistent environmental reservoirs that may have contributed to the persistence of the outbreak.

Taken together, a set of converging epidemiological and microbiological findings supports a potential role of shared shower drains in the persistence of the outbreak. These include the observation of similar contamination patterns across both healthcare facilities, the absence of identifiable direct epidemiological links between several patients within the same wards, frequent patient exposure to shared showers, persistent detection of the epidemic clone in these drains over time, and the high proportion of positive environmental samples originating from shared shower drains. Finally, the interruption of cross-transmission following relocation further supports a role of environmental factors in sustaining transmission.

To better address the question of why shower drains may have contributed to transmission in our specific setting, our interpretation is based on several complementary elements. First, previous reports have documented shower drains and sanitary wastewater systems as potential reservoirs for CPE. Second, discussions with the F-NRC supported the relevance of investigating this hypothesis in the context of our outbreak. Third, although routine environmental cleaning of floors and surfaces had been reinforced, drainage systems were not initially targeted by specific control measures, which may have contributed to the persistence of contamination in these sites.

Drain decontamination has been particularly studied in intensive care units (ICUs) [26]. Daily disinfection is recommended, even outside outbreak settings [27], but recent evidence highlights the limited long-term effectiveness of both chemical and thermal disinfection methods [28]. During outbreaks, drain decontamination remains a major challenge, often requiring prolonged interventions, combinations of multiple disinfection strategies, and, in some cases, complete replacement of the drainage infrastructure [11, 23]. This was reflected in our study: while the control bundles A and B (at RHSM) implemented appeared effective in eliminating drain contamination, no comparable success with bundle C was achieved (UHR, ward F1). In this ward, embedded shared shower drains could not be replaced blocking daily bleaching, and repeated disinfection efforts failed to eradicate the contamination. It is worth noting that ward F1 at UHR was relocated to a newly built surgical ward in early 2025, and no cross-transmission has been detected in the five months since the move (despite readmissions of known carriers). This relocation was the only effective measure to stop the outbreak, further supporting the contribution of shared shower drains in facilitating cross-transmission events.

Our study has several limitations. First, methodological differences existed between the two facilities, reflecting local infection control practices and organisational specificities. Second, not all isolates underwent WGS. However, FTIR-based clustering showed full concordance with WGS results, supporting the robustness of our conclusions. Finally, no environmental follow-up was conducted beyond 2024, precluding assessment of potential long-term persistence or re-emergence events.

Beyond these limitations, our findings have important implications for infection control practices. Wastewater-targeted interventions should not be implemented routinely but rather considered in specific situations, particularly during prolonged outbreaks, when transmission remains uncontrolled despite standard measures, or when cases occur without clear epidemiological links. In such contexts, shared shower drains should be regarded as potential high-risk reservoirs and prioritised for investigation. Depending on local infrastructure and feasibility, a combination of interventions may be required, including restriction of shared shower use, replacement of conventional drains with valve systems, and combined chemical and thermal disinfection strategies. Overall, our results highlight the need for a bundled and context-adapted approach to achieve sustained outbreak control.

## Conclusions

In conclusion, this study underscores the ongoing threat posed by NDM-1-producing Enterobacterale*s* in hospital settings, through the emergence of clonal outbreaks with limited treatment options. The persistence of these high-risk clones, despite classical IPC measures, emphasises the need for reinforced infection control strategies, targeted environmental decontamination protocols, and continuous genomic surveillance. Addressing such outbreaks requires a multidisciplinary approach aligned with the One Health concept, bridging microbiological, environmental, and clinical expertise. These measures should be formally integrated into IPC policies, as their routine application remains inconsistent in current practice.

## Supplementary Information

Below is the link to the electronic supplementary material.

Additional Table 2. MIC values of a subset of NDM-1-producing ECC and CFC clinical isolates from UHR (University Hospital of Rennes) and RHSM (Regional Hospital of Saint-Malo).

Additional Figure S1. Temporal distribution of patients carrying or infected with NDM-producing Enterobacterales (CPE) in the Regional Hospital of Saint-Malo (RHSM) and the University Hospital of Rennes (UHR), 2020–2024. Bars represent the quarterly number of patients colonised or infected with NDM-producing ECC and CFC isolates identified in both hospitals before whole-genome sequencing (WGS) selection. Colors indicate the healthcare facility: blue for RHSM and green for UHR. Each letter within a bar corresponds to a specific ward (A–I): A = Polyvalent Intensive Care Unit; B = Infectious Diseases; C = Haematology; D = Geriatrics; E1 = Hepatology-A; E2 = Hepatology-B; F1 = Digestive Surgery-A; F2 = Digestive Surgery-B; G = Surgical Intensive Care Unit; H = Urology; I = Cardiac Intensive Care Unit.

Additional Fig. 2. FTIR-based clustering of NDM-producing ECC isolates from patients and environmental samples (2020–2024). Hierarchical clustering (Euclidean distance, average linkage) showing relatedness between clinical and environmental (sink and shower drains) isolates collected at the Regional Hospital of Saint-Malo (RHSM) and the University Hospital of Rennes (UHR). The analysis includes both patient and environmental isolates; WGS was performed for all patient isolates and for five representative drain isolates. The cut-off was set at 0.158.

Additional Fig. 3. FTIR-based clustering of NDM-producing Citrobacter freundii complex (CFC) isolates from patients and environmental samples (2023–2024). Hierarchical clustering (Euclidean distance, average linkage) showing relatedness between clinical and environmental (sink and shower drains) isolates collected at the University Hospital of Rennes (UHR). The dataset includes both patient and environmental isolates; WGS was only performed for all patient isolates. The cut-off was set at 0.077.

Additional Table 1. Overview of patient and environmental NDM-producing ECC and CFC isolates included in this study.

## Data Availability

BioProject [PRJNA1353879]. (https:/www.ncbi.nlm.nih.gov/bioproject/PRJNA1353879).

## Abbreviations

CPE: Carbapenemase-producing Enterobacterales
MBLs: Metallo-β-lactamases
ECC: *Enterobacter cloacae* complex
CFC: *Citrobacter freundii *complex
IPC: Infection and prevention control
WGS: Whole genome sequencing
UHR: University Hospital of Rennes
RHSM: Regional hospital of Saint-Malo
MALDI-TOF: Matrix-assisted laser desorption/ionization-time-of flight mass spectrometry
F-NRC: French National Reference Centre
FTIR: Fourier transform infrared
STs: Sequence types
MICs: Minimal inhibitory concentrations
NGS: Next Generation sequencing
SNP: Single-nucleotide polymorphism
MLST: Multilocus Sequence Typing
ICUs: Intensive care units
