# Supplementary material for: Long-term dissemination of NDM-producing Enterobacterales in two French hospitals (2020–2024): outbreak complexity and the role of drains as environmental reservoirs
Source: Antimicrob Resist Infect Control. 2026 Jun 4;15:100. doi: 10.1186/s13756-026-01766-z (PMC13466148; doi:10.1186/s13756-026-01766-z)
Supplement: Supplementary file 1 [file 13756_2026_1766_MOESM2_ESM.pdf]

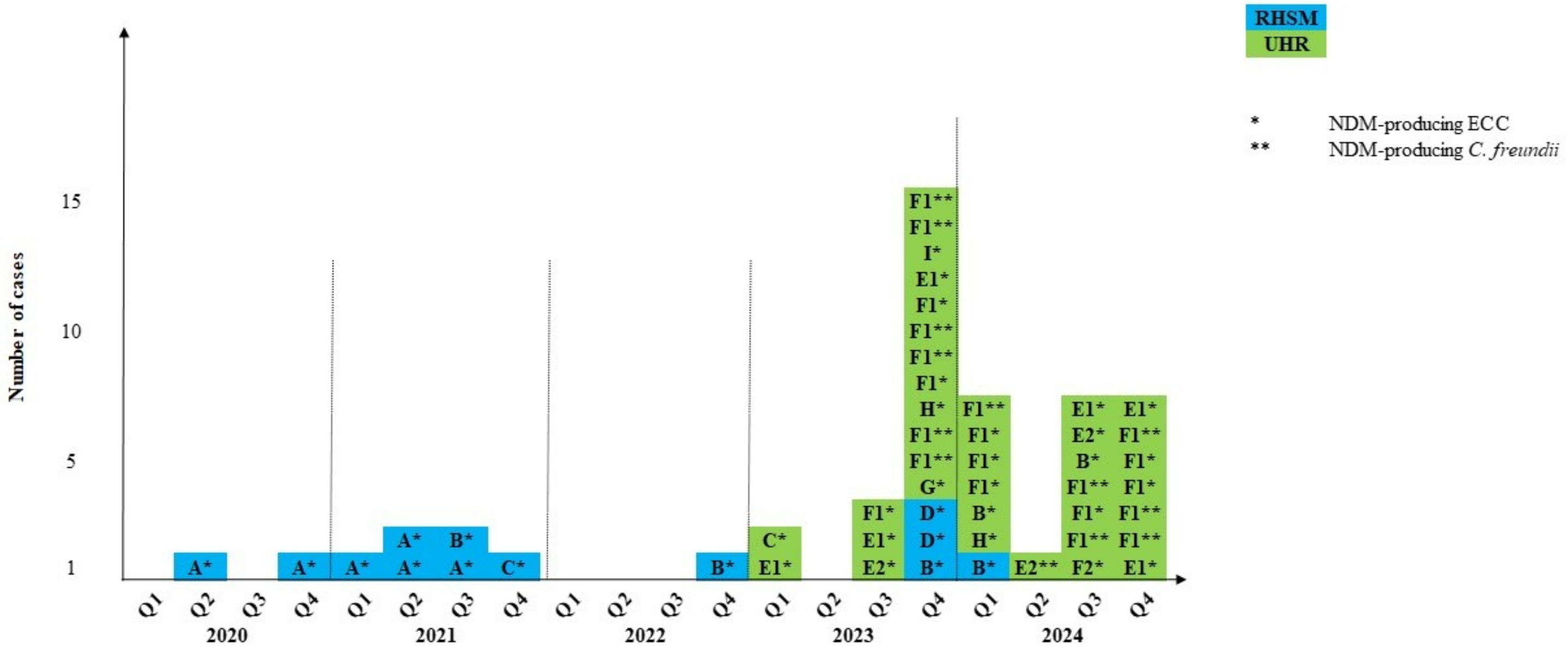

RHSM
UHR
* NDM-producing ECC
** NDM-producing C. freundii
Number of cases
15
10
5
1
A*
A*
A*
A*
A*
B*
A*
C*
B*
C*
E1*
F1*
E1*
E2*
F1**
F1**
I*
E1*
F1*
F1**
F1**
F1*
H*
F1**
F1**
F1**
G*
D*
D*
B*
F1**
F1*
F1*
F1*
B*
H*
B*
E2**
E1*
E2*
B*
F1**
F1*
F1**
F2*
E1*
F1**
F1*
F1*
F1**
F1**
E1*
Q1
Q2
Q3
Q4
2020
2021
2022
2023
2024
