# Supplementary material for: Long-term dissemination of NDM-producing Enterobacterales in two French hospitals (2020–2024): outbreak complexity and the role of drains as environmental reservoirs
Source: Antimicrob Resist Infect Control. 2026 Jun 4;15:100. doi: 10.1186/s13756-026-01766-z (PMC13466148; doi:10.1186/s13756-026-01766-z)
Supplement: Supplementary file 2 [file 13756_2026_1766_MOESM3_ESM.pdf]

WN: **1300-800/cm** Dim. reduction: **PCA (9 PCs / 95.4% variance)** Averaging: **None** Metric/linkage: **Euclidean/Average** Cophenetic correlation coefficient: **0.983** Calculated cut-off: **0.018**

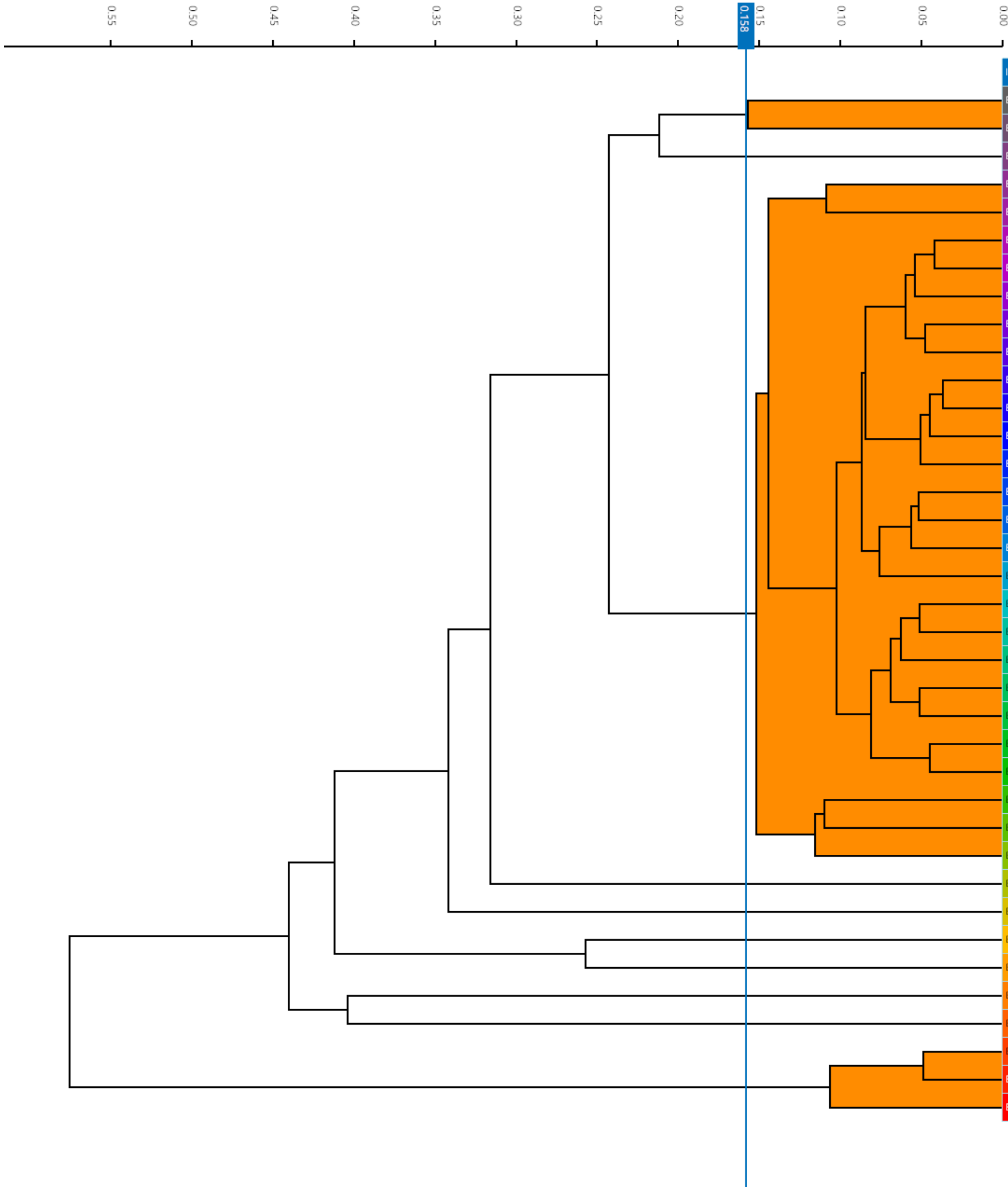

Isolate	ECC_E/P	ECC_CPE	ECC_ST	ECC_Ward	ECC_Facilities	ECC_C/D
EPC-G4-E	Shower drain	NDM	ND	Infectious diseases	RHSM	SS
EPC-F9-E	Sink drain			Emergencies		CS
EPC-G3-E				Polyvalent intensive care unit		R7
EPC-C2-E bis	Shower drain		111	Digestive surgery-A	UHR	SS3648
EPC-C5-P	Patient					R22
EPC-E2-P				Surgery intensive care unit		R-D4
EPC-C1-E	Sink drain			Digestive surgery-A		R11
EPC-F4-P	Patient			Urology		R5
EPC-E1-P				Digestive surgery-A		R14
EPC-B9-E	Shower drain		ND			SS3662
EPC-G6-E bis	Sink drain		111	Polyvalent intensive care unit	RHSM	R4
EPC-D1-P	Patient			Digestive surgery-A	UHR	R22
EPC-F7-P bis				Hepatology-B	RHSM	R224
EPC-D4-P				Digestive surgery-A	UHR	undefined (EPC-D4-P)
EPC-G1-E	Shower drain		ND	Haematology	RHSM	SS
EPC-B7-E	Sink drain			Digestive surgery-A	UHR	SS3662
EPC-G8-E				Geriatrics	RHSM	R443
EPC-G2-E	Shower drain					SS447
EPC-C8-P	Patient		111	Hepatology-A	UHR	R212
EPC-B8-E	Sink drain			Digestive surgery-A		R1
EPC-G7-E			ND	Hepatology RHSM	RHSM	R326
EPC-F8-P	Patient		111	Digestive surgery-A	UHR	R10
EPC-C4-P				Digestive surgery-B		R40
EPC-B1-E bis	Shower drain		ND	Digestive surgery-A		SS3648
EPC-C3-E	Sink drain		111			R10
EPC-E5-P	Patient					R4
EPC-D6-P						R15
EPC-E8-P						undefined (EPC-E8-P)
EPC-B3-E	Sink drain		ND			R25
EPC-B5-E	Shower drain					SS3648
EPC-A9-E	Sink drain	VIM				R12
EPC-A8-E						R11
EPC-F5-P	Patient	NDM	228	Infectious diseases		
EPC-E9-P			269			R6
EPC-A7-E	Sink drain	VIM	ND	Digestive surgery-A		R14
EPC-A2-E						
EPC-B4-E						R26
