# Supplementary material for: Long-term dissemination of NDM-producing Enterobacterales in two French hospitals (2020–2024): outbreak complexity and the role of drains as environmental reservoirs
Source: Antimicrob Resist Infect Control. 2026 Jun 4;15:100. doi: 10.1186/s13756-026-01766-z (PMC13466148; doi:10.1186/s13756-026-01766-z)
Supplement: Supplementary file 3 [file 13756_2026_1766_MOESM4_ESM.pdf]

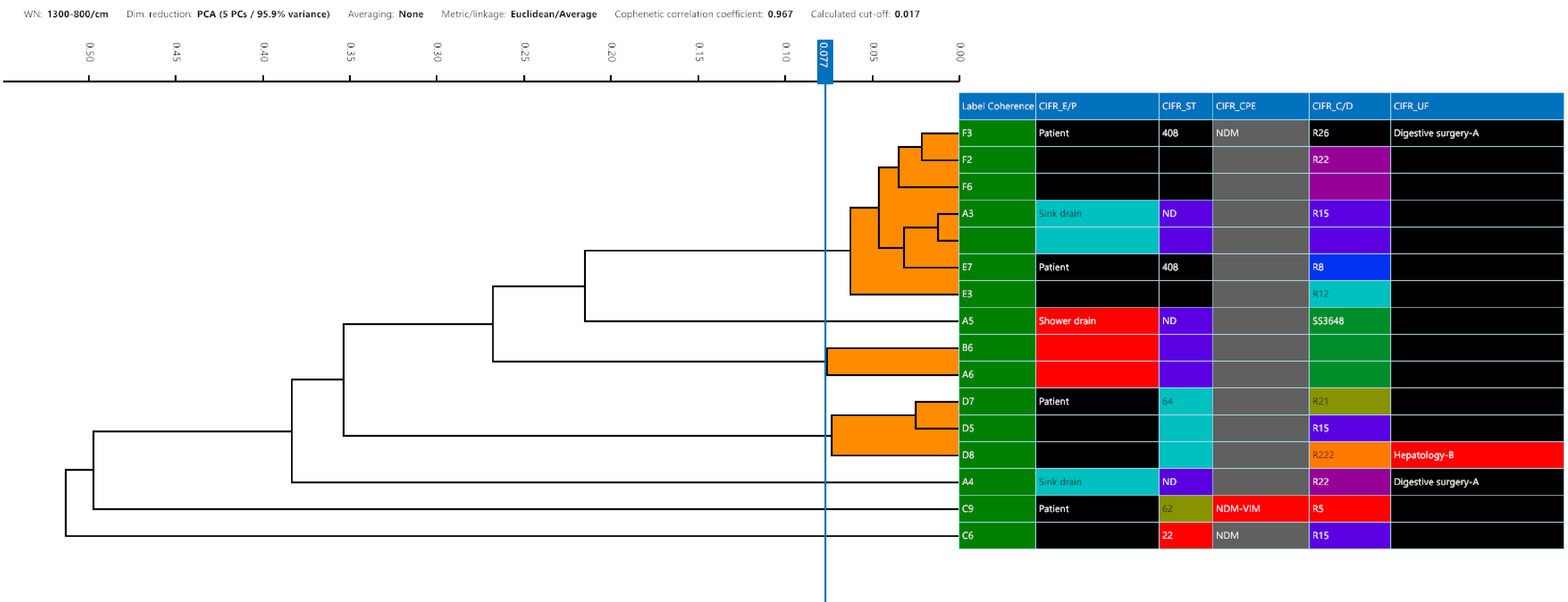
WN: 1300-800/cm
Dim. reduction: PCA (5 PCs / 95.9% variance)
Averaging: None
Metric/linkage: Euclidean/Average
Cophenetic correlation coefficient: 0.967
Calculated cut-off: 0.017
0.50
0.45
0.40
0.35
0.30
0.25
0.20
0.15
0.10
0.077
0.05
0.00
Label Coherence
CIFR_E/P
CIFR_ST
CIFR_CPE
CIFR_C/D
CIFR_UF
F3
Patient
408
NDM
R26
Digestive surgery-A
F2
R22
F6
A3
Sink drain
ND
R15
E7
Patient
408
R8
E3
R12
A5
Shower drain
ND
SS3648
B6
A6
D7
Patient
64
R21
D5
R15
D8
R222
Hepatology-B
A4
Sink drain
ND
R22
Digestive surgery-A
C9
Patient
62
NDM-VIM
R5
C6
22
NDM
R15
